# Cancer cell progression and chemoimmunotherapy--dual effects in the induction of resistance to therapy.

**DOI:** 10.1038/bjc.1996.82

**Published:** 1996-02

**Authors:** J. Hamuro, T. Kikuchi, F. Takatsuki, M. Suzuki

**Affiliations:** Basic Research Laboratories, Central Research Laboratories, Ajinomoto Co., Kawasaki, Japan.

## Abstract

To determine whether resistance to chemoimmunotherapy is acquired during therapy, we investigated the effects of chemotherapeutic agents and anti-tumour polysaccharide, lentinan, on the progression of Rous sarcoma virus-induced S908.D2 fibrosarcomas. The chemoimmunotherapy was effective against the parental S908.D2-bearing mice. Nearly all the mice that were treated with cyclophosphamide (CY) and lentinan achieved complete tumour regression. Only a few of the mice that achieved complete regression of the primary tumours showed a recurrence of the tumour in regional lymph nodes. S908.D2-vp.1 was established from metastatic tumours that developed in the regional lymph nodes of parental S908.D2-bearing mice during therapy. S908.D2-vp.2-or vp.3 cells were sequentially derived in a similar way from S908.D2-vp.1-or-vp.2-bearing mice respectively, in which complete tumour regression at each primary site was achieved during therapy. These lines acquired resistance to CY and lentinan and also to 5-fluorouracil (5-FU)/5'-deoxy-5-fluorouracil and lentinan. No significant difference in either the sensitivity to 5-FU or 4-deoxycyclophosphamide in vitro or in the susceptibility to immune effector cells was observed between the parental and progressed lines (S908.D2-vp1 -vp3). There was an increase in the level of prostaglandin E2 (PGE2) in the progressed lines during repeated therapy (parental, 1171 pg ml(-1); vp.1, 2199 pg ml(-1); vp.2, 5500pg ml(-1); vp3, 16187 pg ml(-1)). There was no significant increase in the production of transforming growth factor beta (TGF-beta). The amount of interleukin-2 (IL-2) produced by spleen cells isolated from the S908.D2-vp.2-bearing mice was decreased compared with the amount produced by the parental S908.D2- bearing mice. Furthermore, combination therapy with lentinan and IL-2 achieved complete tumour regression in all the mice transplanted with S908.D2 progressed tumour lines, although IL-2 alone did not show any anti-tumour effects in either the S908.D2 parental or progressed lines. The findings suggest that the reduced production of IL-2 induced an increase in the production of the PGE2 by progressed tumour lines is involved in the acquisition of resistance.


British Journal of Cancer (1996) 73, 465-471
© 1996 Stockton Press All rights reserved 0007-0920/96 $12.00

### Cancer cell progression and chemoimmunotherapy – dual effects in the induction of resistance to therapy

J Hamuro, T Kikuchi, F Takatsuki and M Suzuki

*Basic Research Laboratories, Central Research Laboratories, Ajinomoto Co., Kawasaki, 210, Japan.*

**Summary** To determine whether resistance to chemoimmunotherapy is acquired during therapy, we investigated the effects of chemotherapeutic agents and anti-tumour polysaccharide, lentinan, on the progression of Rous sarcoma virus-induced S908.D2 fibrosarcomas. The chemoimmunotherapy was effective against the parental S908.D2-bearing mice. Nearly all the mice that were treated with cyclophosphamide (CY) and lentinan achieved complete tumour regression. Only a few of the mice that achieved complete regression of the primary tumours showed a recurrence of the tumour in regional lymph nodes. S908.D2-vp.1 was established from metastatic tumours that developed in the regional lymph nodes of parental S908.D2-bearing mice during therapy. S908.D2-vp.2-or vp.3 cells were sequentially derived in a similar way from S908.D2-vp.1-or-vp.2-bearing mice respectively, im which complete tumour regression at each primary site was achieved during therapy. These lines acquired resistance to CY and lentinan and also to 5-fluorouracil (5-FU)/5'-deoxy-5-fluorouracil and lentanin. No significant difference in either the sensitivity to 5-FU or 4-deoxycyclophosphamide *in vitro* or in the susceptibility to immune effector cells was observed between the parental and progressed lines (S908.D2-vp.1 ~ -vp.3). There was an increase in the level of prostaglandin $E_2$ ($PGE_2$) in the progressed lines during repeated therapy (parental, 1171 pg $ml^{-1}$; vp.1, 2199 pg $ml^{-1}$; vp.2, 5500 pg m $l^{-1}$; vp3, 16187 pg $ml^{-1}$). There was no significant increase in the production of transforming growth factor $\beta$ (TGF-$\beta$). The amount of interleukin-2 (IL-2) produced by spleen cells isolated from the S908.D2-vp.2-bearing mice was decreased compared with the amount produced by the parental S908.D2-bearing mice. Furthermore, combination therapy with lentinan and IL-2 achieved complete tumour regression in all the mice transplanted with S908.D2 progressed tumour lines, although IL-2 alone did not show any anti-tumour effects in either the S908.D2 parental or progressed lines. The findings suggest that the reduced production of IL-2 induced an increase in the production of the $PGE_2$ by progressed tumour lines is involved in the acquisition of resistance.

**Keywords:** Tumour progression; chemoimmunotherapy; prostaglandin; lentinan; interleukin 2

The genetic instability and heterogeneity of tumour cells are among the most serious obstacles in the treatment of cancer patients. Multiple genomic changes in tumour cells are not only required for the full development of tumour phenotyes but also for the progression of tumours, including the acquisition of metastatic traits and resistance to therapeutic treatments (Vogelstein *et al.*, 1989; Hunter, 1991; Chang and Loeb, 1993). It has been suggested that oxygen radicals produced by carcinogenic substances and radiation are involved in the acquisition of genetic instability (Zimmarman and Cerutti, 1984; Turver and Brown, 1987; Ward, 1988). Recent studies have shown that anti-cancer drugs themselves can induce tumour progression even as they work to destroy tumour cells (McMillan and Hart, 1987; Imamura *et al.*, 1990). The resistance to chemotherapeutic agents caused by genetic changes in tumour cells is one of the factors involved in tumour progression (Goldie and Coldman, 1979). Studies to elucidate the mechanisms underlying the acquisition of resistance to drugs have revealed that many genes, such as *MDRI* and *GST-π*, are involved in this process (Kramer *et al.*, 1988; Bradly *et al.*, 1988; Gottesman 1988. Genetic changes in tumour cells can also result in the escape of tumour cells from surveillance by host immune system (Johnson *et al.*, 1989; Tanaka and Tevethia, 1988; Doherty *et al.*, 1984). Such genetic changes include the loss of target proteins (tumour-associated antigens), which are usually recognised by antibodies or cytotoxic T lymphocytes (CTLs), from the surface of tumour cells, and a decrease in the expression of major histocompatibility complex (MHC) class I antigens on tumour cells (Doherty *et al.*, 1984; Tanaka and Tevethia, 1988).

Correspondence: M Suzuki, Basic Research Laboratories, Central Research Laboratories, Ajinomoto Co., 1-1, Suzuki-cho, Kawasaki-ku, Kawasaki, 210, Japan
Received 11 April 1995; revised 8 June 1995; accepted 12 September 1995

Recently, many attempts to improve the therapeutic efficacy of anti-cancer drugs have combined the use of chemotherapeutic and immunotherapeutic agents (chemoimmunotherapy). Synergistic effects resulting from such a combination of treatments have been demonstrated in experimental models and in clinics (Paciucci *et al.*, 1989; Mitchell, 1992). We have also observed synergistic anti-tumour effects against an established murine fibrosarcoma after treatments with chemotherapeutic agents and an anti-tumour polysaccharide, lentinan (Suzuki *et al.*, 1994). Lentinan is a true biological response modifier in the sense that it lacks direct cytotoxic effects against tumour cells and is used in combination with tegafur for the clinical treatment of gastric cancer patients in Japan (Taguchi *et al.*, 1985). It is not known whether the acquisition of resistance to therapy occurs during chemoimmunotherapy, or whether the mechanisms underlying the acquisition of resistance are distinct from those induced by chemotherapeutic agents or immune effector cells alone.

In this paper we demonstrate that the acquisition of resistance to therapy does occur during chemoimmunotherapy using cyclophosphamide (CY) and lentinan in a murine fibrosarcoma system. The possible mechansims that lead to resistance to chemoimmunotherapy were also investigated.

### Materials and methods

### *Mice*

B10.D2 mice were purchased from Shizuoka Laboratory Animal Center (Shizuoka, Japan). The mice were maintained in specific pathogen-free conditions. Normal female mice, 6–10 weeks of age, were used for the experiments.

### *Tumour and target cells for cell-mediated cytotoxicity assay*

C57BL/6-derived EL-4 thymoma and A/Sn-derived Moloney virus-induced Yac-1 lymphoma were maintained by culture *in*

vitro in RPMI-1640 medium that was supplemented with 5% fetal bovine serum (FBS). B10.D2-derived Rous sarcoma virus (RSV)-induced S908.D2 sarcoma, which was a kind gift from Dr S Fujimoto of Kochi Medical College, was maintained by culture *in vitro* in RPMI-1640 medium that was supplemented with 5% FBS. For evaluation of anti-tumour activity, $2 \times 10^6$ S908.D2 cells in 0.1 ml of saline were inoculated intradermally (i.d.) into syngeneic hosts. The S908.D2-vp.1 subline was isolated from metastatic tumour cells obtained from the regional lymph nodes of the parental S908.D2-bearing mice during combination therapy with CY and lentinan. S908.D2-vp.2 or -vp.3 were isolated in the same way from metastatic tumour cells from the regional lymph nodes of mice that had been transplanted with S908.D2-vp.1 or -vp.2 respectively. The S908.D2-nt.1 subline was isolated from metastatic tumour cells from the regional lymph nodes of mice that had been transplanted with the parental S908.D2 and received saline only. S908.D2-nt.2 or -nt.3 were isolated in the same way from mice that had been transplanted with S908.D2-nt.1 or -nt.2 respectively. These S908.D2 sublines were maintained for at least 6 months in RPMI-1640 medium that was supplemented with 5% FBS.

*Reagents*

Cyclophosphamide (CY), 5-fluorouracil (5-FU) and 5'-deoxy-5-fluorouracil (5'-DFUR) were obtained from Shionogi Pharmaceutical Co. (Osaka, Japan), Kyowa Hakko, (Tokyo, Japan) and Nippon Roche (Tokyo, Japan) respectively. 4-Deoxy cyclophosphamide was a kind gift from Dr T Yoshioka of Shionogi Pharmaceutical Co. Poly-I:C was obtained from Sigma (St Louis, MO, USA.). Recombinant murine interferon-$\gamma$ (IFN-$\gamma$) was obtained from Genzyme (Cambridge, MA, USA) and its specific activity was $1 \times 10^7$ units $mg^{-1}$. Recombinant human interleukin-2 (IL-2) was prepared as described elsewhere (Satoh *et al.*, 1987) and its specific activity was $5 \times 10^7$ units $mg^{-1}$. Lentinan, a fully purified $\beta$-1,3-glucan with $\beta$-1,6 branches obtained from *Lentinus edodes*, was prepared as described elsewhere (Chihara *et al.*, 1969).

*Evaluation of anti-tumour activity*

Approximately $2 \times 10^6$ S908.D2 lines were inoculated i.d. into B10.D2 mice. For chemoimmunotherapy either 100 mg $kg^{-1}$ CY, 75 mg $kg^{-1}$ 5-FU, 130 mg $kg^{-1}$ 5'DFUR or saline was administered intraperitoneally (i.p.) into mice 10 days after tumour inoculation. Seven days after the injection of a chemotherapeutic agent, 5 mg $kg^{-1}$ lentinan or saline was administered i.p. daily for five consecutive days. For combined immunotherapy, 5 mg $kg^{-1}$ lentinan or saline was administered i.p. into mice daily for 4 days starting 10 days after tumour inoculation. After the final day of injection of lentinan, 0.1 mg $kg^{-1}$ (2 $\mu$g per mouse) IL-2 or saline was administered i.p. twice a day for 4 days. Therapeutic activity was evaluated by the inhibition of tumour growth and the prolongation of survival days. The size of each tumour was represented as the product of the largest tumour diameter and the shortest diameter ($mm^2$).

In vitro *susceptibility of S908.D2 lines against chemotherapeutic agents*

Approximately $2 \times 10^4$ cells per well of each S908.D2 line were cultured in 96-well flat-bottom culture plates with various concentrations of 5-FU or 4-deoxycyclophosphamide. After 48 h culture, cell growth was examined by an MTT assay as described elsewhere (Mosmann, 1983).

*Preparation of cytotoxic T lymphocytes (CTLs) against S908.D2*

B10.D2 mice were immunised i.p. twice with $5 \times 10^6$ cells of an 88 Gy irradiated S908.D2 parental line at an interval of 2 weeks. One week after the final immunisation, spleen cells were prepared from the immunised mice and cultured for 5 days with an 88 Gy irradiated S908.D2 parental line (responder/stimulator (R/S) ratio = 300). The cytotoxic activity against each S908.D2 line was assayed in a $^{51}Cr$-release assay (effector/target (E/T) = 100). The percentage of specific lysis was calculated by the standard formula: % specific lysis = (experimental release/spontaneous release) (maximum release/spontaneous release)$^{-1}$ $\times$ 100.

*Preparation of activated natural killer (NK) and lymphokine-activated killer (LAK) cells*

Approximately $5 \times 10^6$ cells $ml^{-1}$ of spleen cells were cultured with 100 $\mu$g $ml^{-1}$ poly-I:C for 24 h or with 250 units $ml^{-1}$ rIL-2 for 3 days. The resultant cells were used as NK or LAK cells respectively. The cytotoxic activity against each S908.D2 line was assayed in a $^{51}Cr$-release assay (E/T = 100) as described above.

*Preparation of activated macrophages*

Approximately $1 \times 10^5$ cells per well of peritoneal cells were cultured in 96-well flat-bottom culture plates with 100 units $ml^{-1}$ murine IFN-$\gamma$ for 24 h and the resultant cells were used as activated macrophages. The activated macrophages and each S908.D2 subline were mixed (E/T = 12.5) and cultured for 24 h. Cytostatic activity was examined by incorporation of tritiated thymidine ([$^3H$] TdR) during the last 4 h of the culture. The percentage of specific inhibition was calculated by the standard formula:% specific inhibition = (incorporation of [$^3H$]TdR by tumour cells cultured with macrophages) (incorporation of [$^3H$] TdR of tumour cells cultured without macrophages)$^{-1}$ $\times$ 100.

*Flow cytometric analysis*

Each S908.D2 subline was stained with H-2K$^d$-specific antiserum (Meiji Nyugyo, Tokyo) or H-2D$^d$-specific antiserum (Meiji Nyugyo) and FITC-conjugated mouse Ig-specific antiserum (Cedarlane, Ontario, Canada). The fluorescence intensity was measured by FACScan (Becton Dickinson, Mountain View, CA, USA).

*Assay for prostaglandin $E_2$ ($PGE_2$)*

Approximately $1 \times 10^4$ cells $ml^{-1}$ of each S908.D2 line were cultured for 96 h in RPMI-1640 medium that was supplemented with 10% FBS. The cells were removed and the medium was harvested and stored at $-110$°C until needed for the $PGE_2$ assay. The amount of $PGE_2$ was measured using a commercially available enzyme immunoassay kit (Amersham, Buckinghamshire, UK) according to the manufacturer's protocol.

*Assay for transforming growth factor $\beta$* (TGF-$\beta$)

Approximately $1 \times 10^4$ cells $ml^{-1}$ of each S908.D2 line were cultured in RPMI-1640 medium that was supplemented with 10% FBS. After 96 h the culture medium was replaced with fresh RPMI-1640. After 24 h of culture, the cells were removed and the conditioned medium was harvested and heat-treated at 85°C for 10 min to convert latent forms of TGF-$\beta$ into active ones. The resultant samples were stored at $-110$°C until needed for the TGF-$\beta$ assay. Mv1Lu cells for the TGF-$\beta$ assay were a kind gift from Dr H Fujiwara of Osaka University. The growth inhibition assay for TGF-$\beta$ was performed according to the method described elsewhere (Cheifetz *et al.*, 1987). Briefly, Mv1Lu cells ($1 \times 10^{4)}$ )were cultured for 24 h in 96-well flat-bottom plates with diluted samples or with control recombinant TGF-$\beta$ (King Syuzo Co., Hyogo, Japan) in RPMI-1640 medium that was supplemented with 5% FBS. Proliferation was assessed by determining the uptake of [$^3H$]TdR during a 6 h pulse with 37 kBq of [$^3H$]TdR per well.

*Production of IL-2 and assay for IL:-2 activity*

Spleen cells ($3 \times 10^6$ ml$^{-1}$) from either the S908.D2 parental- or vp.2-bearing B10.D2 mice were cultured with irradiated S908.D2 parental or vp.2 cells ($1 \times 10^4$ ml$^{-1}$) in RPMI-1640 medium that was supplemented with 10% FBS. After incubation for 2 days, culture supernatants were harvested and stored at −20°C until use. Supernatants were assayed for IL-2 activity using an IL-2-dependent T cell line, CTLL-2. CTLL-2 ($4 \times 10^3$ per well) were cultured with the supernatants for 24 h. Proliferation was assessed by determining the uptake of [$^3$H]TdR during a 4 h pulse with 18.5 kBq [$^3$H]TdR per well.

### Results

*Acquisition of resistance to chemoimmunotherapy in progressed lines*

To evaluate the effect of chemoimmunotherapy on tumour progression, a B10.D2-derived RSV-induced fibrosarcoma, S908.D2, was used. S908.D2 was chosen because it exhibits a slow rate of growth in syngeneic hosts and a resistance to chemotherapeutic agents. Table I shows the results of the anti-tumour effects of chemoimmunotherapy against S908.D2. The B10.D2 mice were inoculated i.d. with $2 \times 10^6$ viable S908.D2 tumour cells. CY (100 mg kg$^{-1}$), 5-FU (75 mg kg$^{-1}$) or 5'-DFUR (130 mg kg$^{-1}$) was administered i.p. 10 days after tumour inoculation. One week after the chemotherapy, 5 mg kg$^{-1}$ lentinan was administered i.p. for 5 consecutive days. CY, 5-FU or 5'-DFUR alone exerted only a marginal effect on the inhibition of the growth of the tumour. Only 3 of 20 mice treated with lentinan alone showed complete regression (experiments 1–3). Although higher doses of CY (200 mg kg$^{-1}$) or 5-FU (150 mg kg$^{-1}$) alone augmented extent of the growth inhibition of the tumour, no mice that received the therapy entered into complete remission (data not shown). Treatment with higher doses of lentinan (15 mg kg$^{-1}$) alone could not augment the anti-tumour effects. The combination of lentinan and a chemotherapeutic agent (CY, 5-FU or 5'-DFUR) exerted synergistic anti-tumour effects and resulted in a complete regression of the tumour in all mice. Almost all of the mice treated with lentinan and CY appeared to be completely cured (>250 days survival), and only a few showed tumour recurrence in regional lymph nodes (around 10% of the mice). The metastatic tumours were excised and cultured *in vitro*. The established subline was designated S908.D2-vp.1. S908.D2-vp.2 or -vp.3 sublines were sequentially derived in a similar way from S908.D2-vp.1- or -vp.2-bearing mice respectively, in which complete tumour regression at each primary site was observed during therapy. The S908.D2-nt.1 subline was established from metastatic tumours from the regional lymph nodes of parental S908.D2-bearing mice treated with saline only. S908.D2-nt.2 or -nt.3 were sequentially derived in the same way from mice that had been transplanted with S908.D2-nt.1 or -nt.2. The therapeutic efficacy of chemoimmunotherapy against these S908.D2 sublines was examined. Only 60%, 28.5%, or 14.2% of mice inoculated with S908.D2-vp.1 or -vp.2 or -vp.3 respectively, entered into complete remission after treatment with lentinan and CY (Table II). In contrast, the chemoimmunotherapy resulted in a complete regression of the tumour in all mice inoculated with S908.D2-nt.1 or -nt.2 or -nt.3 (Table II), indicating that these sublines did not acquire resistance to the chemoimmunotherapy. Hereafter, the S908.D2-vp.1, -vp.2, -vp.3 cell lines are referred to as progressed lines.

**Table I** Anti-tumour effects of chemotherapeutic drugs and/or lentinan against S908.D2 fibrosarcoma in B10.D2 mice

| *Treatments* | *Tumour size*[a] | *Complete regression*[b] |
|---|---|---|
| Experiment 1 | | |
| Control | 186.4±31.7 | 0/7 |
| CY | 101.7±18.1 | 0/6 |
| LNT | 167.1±17.3 | 0/7 |
| CY/LNT | 4.1±10.2 | 6/6 |
| Experiment 2 | | |
| Control | 124.8±16.8 | 0/7 |
| 5-FU | 118.6±27.8 | 0/7 |
| LNT | 109.4±34.4 | 1/7 |
| 5-FU/LNT | 48.7±28.0 | 7/7 |
| Experiment 3 | | |
| Control | 164.6±21.8 | 0/6 |
| 5'-DFUR | 126.5±30.1 | 0/6 |
| LNT | 78.9±64.7 | 2/6 |
| 5'-DFUR/LNT | 0.0±0.0 | 7/7 |

Approximately $2 \times 10^6$ cells from the S908.D2 tumour were inoculated i.d. on day 0. The mice were treated with saline, cyclophosphamide (CY) (100 mg kg$^{-1}$), 5-FU (75 mg kg$^{-1}$), or 5'-DFUR (130 mg kg$^{-1}$, i.p.) on day 10 and treated with saline or lentinan (LNT) (5 mg kg$^{-1}$, i.p.) on days 17–21. [a]Tumour size was represented as the product of the largest tumour diameter and the shortest tumour diameter (mm$^2$) and evaluated on day 28 (experiment 1), day 35 (experiment 2) or day 32 (experiment 3). [b]Complete regression at original site was defined when tumour had not regrown for more than 60 days.

*Characterisation of the progressed lines in comparison with the parental line*

The growth rates *in vitro* and *in vivo* of the progressed lines were compared with the growth rates of the parental line. No increase in the *in vivo* growth rate was observed in the progressed lines (data not shown). The rate of growth *in vivo* of the progressed lines was slightly less than that of the parental line (data not shown).

We investigated whether the acquired resistance of the progressed lines to the combination therapy of lentinan and CY was due to a decreased sensitivity to CY. S908.D2-vp.3 also acquired resistance to the combination of 5-FU/lentinan and 5'-DFUR/lentinan (data not shown and Table III). There was no difference between the parental line and progressed lines in their sensitivity *in vitro* to 5-FU or 4-deoxy-cyclophosphamide, which is the active form of CY (Figure 1). Furthermore, lentinan did not affect the *in vitro* growth of either the parental or progressed lines (data not shown).

To determine whether the acquired resistance of the progressed lines to chemoimmunotherapy was due to a decrease in the susceptibility of tumour cells to immune effector cells, the susceptibility of S908.D2 lines to CTLs, NK cells, LAK cells or activated macrophages was examined. As shown in Table IV, CTLs raised against an S908.D2 parental line were equally cytotoxic against S908.D2 parental and progressed lines. NK cells and LAK cells were induced from B10.D2 spleen cells by culture with 100 μg ml$^{-1}$ poly I:C or with 250 u ml$^{-1}$ human rIL-2 respectively. S908.D2 parental

**Table II** Decreased therapeutic effects of combination therapy with CY and lentinan

| *S908.D2 lines* | *Tumour size*[a] | *Complete regression*[b] |
|---|---|---|
| Parental | 0.0±0.0 (day 40) | 5/5 |
| vp.1 | 84.3±143.0 (day 42) | 3/5 |
| vp.2 | 86.5±88.5 (day 38) | 2/7 |
| vp.3 | 288.9±147.0 (day 41) | 1/7 |
| nt.1 | 3.7±11.5 (day 42) | 10/10 |
| nt.2 | 0.0±0.0 (day 38) | 9/9 |
| nt.3 | 0.0±0.0 (day 40) | 9/9 |

[a]Tumour size was represented as the product of the largest tumour diameter and the shortest tumour diameter (mm$^2$) and evaluated around day 40. [b] Complete regression at original site was defined when tumour had not regrown for more than 80 days. Approximately $2 \times 10^6$ cells from the S908.D2 sublines were inoculated i.d. on day 0. The mice were treated with CY (100 mg kg$^{-1}$, i.p.) on day 10 and lentinan on days 17–21 (5 mg kg$^{-1}$, i.p.).

**Table III** Anti-tumour effects of 5'-DFUR and lentinan against the progressed line, S908.D2-vp.3, in B10.D2 mice

| *Treatment* | *Tumour size*[a] | *Complete regression*[b] |
|---|---|---|
| Control | 159.8 ± 40.0 | 0/7 |
| 5'-DFUR | 162.1 ± 48.5 | 0/9 |
| 5'-DFUR/LNT | 129.2 ± 38.3 | 0/9 |

[a]Tumour size was represented as the product of the largest tumour diameter and the shortest tumour diameter ($mm^2$) and evaluated on day 27. [b] Complete regression at original site was defined when tumour had not regrown for more than 60 days. Approximately $2 \times 10^6$ cells from the S908.D2-vp.3 tumour were inoculated i.d. on day 0. The mice were treated with saline or 5'-DFUR (130 mg $kg^{-1}$, i.p.) on day 10 and saline or lentinan (5 mg $kg^{-1}$, i.p.) on days 17–21.

**Figure 1** *In vitro* susceptibility to chemotherapeutic drugs of S908.D2 parental and progressed lines. Approximately $2 \times 10^4$ cells per well of each S908.D2 line were cultured in 96-well flat-bottom culture plates with various concentrations of 5-FU (**a**) or 4-deoxycyclophosphamide (4-HCY) (**b**). After 48 h culture cell growth was examined by MTT assay. One hundred per cent cell growth indicated the average of each cell growth cultured with medium alone. Cell lines: S908.D2 parental (●); vp.1 (▲); vp.2 (○); and vp.3 (□).

and progressed lines were both relatively resistant to poly I:C-activated NK cells and no significant differences in susceptibility were observed between parental and progressed lines (Table IV). In contrast, both lines were highly sensitive to LAK cells. Again, no significant differences in susceptibility to LAK cells were observed (Table IV). Activated macrophages were induced from B10.D2 peritoneal cells by culture with 100 u $ml^{-1}$ murine IFN-γ. No significant differences in susceptibility of these lines to activated macrophages were observed (Table IV).

The parental lines also expressed relatively high levels of class I H-2 antigens (H-2K[d] and H-2 D[d]), and no reduction in the expression of H-2 antigens was observed in progressed lines (data not shown).

*$PGE_2$ and TGF-β production in S908.D2 parental and the progressed lines*

The results shown in Table IV indicate that both S908.D2 parental and progressed lines were antigenic in immunocompetent syngeneic hosts. Since immunosuppression caused by immunosuppressive factor(s) produced by tumour cells has been commonly observed in experimental models or in clinics (Tada *et al.*, 1991; Balch *et al.*, 1984), we determined whether the acquired resistance was due to an increase in the

**Table IV** Target susceptibility of S908.D2 parental and the progressed lines to immune effector cells

| | *Effectors* | | | |
|---|---|---|---|---|
| *Targets* | *NK*[a] | *LAK*[b] | *CTLs*[c] | *Activated macrophages*[d] |
| S908.D2 parental | 22.7 ± 3.5 | 51.5 ± 0.2 | 35.0 ± 3.8 | 93.4 ± 6.5 |
| S908.D2 vp.1 | 9.3 ± 5.2 | 53.1 ± 5.6 | 37.1 ± 0.8 | 82.1 ± 6.3 |
| S908.D2 vp.2 | 21.8 ± 6.3 | 71.4 ± 2.1 | 40.0 ± 3.4 | 85.2 ± 4.5 |
| S908.D2 vpS3 | 23.4 ± 10.5 | 56.5 ± 6.7 | 37.7 ± 11.3 | 91.2 ± 1.5 |

[a]Approximately $5 \times 10^6$ $ml^{-1}$ spleen cells were cultured with 100 μg $ml^{-1}$ poly I:C for 24 h. Killing activity was examined by a 4 h $^{51}Cr$-release assay, E/T = 100. Killing activity of the NK cells against NK-sensitive RL-male 1 cells was 54.7%. [b]Approximately $5 \times 10^6$ $ml^{-1}$ spleen cells were cultured with 250 u $ml^{-1}$ IL-2 for 3 days. Killing activity was examined by a 4 h $^{51}Cr$-release assay, E/T = 100. Killing activity of the LAK cells against LAK-sensitive EL-4 cells was 30.1%. [c]Approximately $3 \times 10^6$ $ml^{-1}$ spleen cells from B10.D2 mice immunised with S908.D2 parental lines were cultured with $1 \times 10^4$ $ml^{-1}$ irradiated S908.D2 parental cells as stimulator for 5 days. Killing activity was examined by a 4 h $^{51}Cr$-release assay, E/T = 100. [d]Approximately $1 \times 10^6$ $ml^{-1}$ peritoneal cells were cultured with 100 u $ml^{-1}$ IFN-γ for 24 h. Cytostatic activity was examined by incorporation of tritiated thymidine for 4 h, E/T = 12.5.

**Table V** TGF-β and $PGE_2$ production by S908.D2 parental and progressed lines

| *Tumour lines* | *TGF-β*[a] | *$PGE_2$*[b] |
|---|---|---|
| S908.D2 parental | 480 ± 10[c] | 1171 ± 15[d] |
| S908.D2-vp.1 | 170 ± 100 | 2199 ± 137 |
| S908.D2-vp.2 | 130 ± 190 | 5500 ± 750 |
| S908.D2-vp.3 | 290 ± 40 | 16187 ± 8812 |
| *P*-value[e] | > 0.05 | < 0.05 |

[a]Approximately $1 \times 10^4$ cells $ml^{-1}$ each S908.D2 line were cultured with RPMI-1640 supplemented with 10% FBS for 96 h. The medium was removed and cells were further cultured in serum-free RPMI for 24 h. The cell-free conditioned medium was harvested for the TGF-β assay. The medium for TGF-β assays was heat-treated at 85°C for 10 min. [b]Approximately $1 \times 10^4$ cells $ml^{-1}$ each S908.D2 line were cultured with RPMI-1640 supplemented with 10% FBS for 96 h and the medium was harvested for the $PGE_2$ assay. [c]The amount of TGF-β (pg $ml^{-1}$) in the medium was measured by a bioassay using Mv1Lu. [d]The amount of $PGE_2$ (pg $ml^{-1}$) in the medium was measured by an enzyme immunoassay kit. [e]The statistical significance of the differences of TGF-β and $PGE_2$ production between cell lines was evaluated by the Kruskal–Wallis test.

production of immunosuppressive factor(s). The production of TGF-$\beta$ and $PGE_2$, the immunosuppressive factors known to be produced by tumour cells (Ceuppens and Goodwin, 1981; Kehrl *et al.*, 1986), was examined in S908.D2 parental and progressed lines. No significant increase in the production of TGF-$\beta$ was observed among these lines (Table V). In contrast, the levels of $PGE_2$ in progressed lines increased relative to levels in the parental line during repeated chemoimmunotherapy ($P<0.05$ by the Kruskal–Wallis test). The parental line produced relatively low levels of $PGE_2$ (Table V). Elevated levels of $PGE_2$ were not observed in S908.D2-nt.1 (1500 pg $ml^{-1}$), -nt.2 (300 pg $ml^{-1}$) or -nt.3 (1500 pg $ml^{-1}$).

### *Effects of combination therapy with lentinan and IL-2 on progressed lines*

Defects in IL-2/IL-2 receptor systems are considered one of the mechanisms of immunosuppression caused by $PGE_2$ (Krause and Deutsch, 1991; Rappaport and Dodge, 1982; Parhar and Lala, 1987). To determine whether an increase in the production of $PGE_2$ in the progressed lines could result in defects in IL-2/IL-2 receptor systems, the amount of IL-2 present in culture supernatants after mixed lymphocyte tumour cultures (MLTCs) was determined using spleen cells from S908.D2 parental- or S908.D2-vp.2-bearing mice. The amount of IL-2 in supernatants from MLTCs using spleen

**Figure 2** Anti-tumour effects of lentinan and IL-2 against the tumour cell line S908.D2-vp.2 in B10.D2 mice. Approximately $2\times10^6$ cells from the S908.D2-vp.2 tumour were inoculated i.d. on day 0. The growth curves of each tumour in mice treated with saline (i.p.) on days 10–13 and days 14–17 (**a**), mice treated with lentinan (5 mg $kg^{-1}$, i.p.) on days 10–13 (**b**), IL-2 (0.1 mg $kg^{-1}$ twice per day, i.p.) on days 14–17 (**c**), and mice treated with both lentinan and IL-2 (**d**). Tumour size was taken to be the product of the largest tumour diameter and the shortest tumour diameter ($mm^2$). Each line represents the relative tumour size in the individual mice.

cells from S908.D2-vp.2-bearing mice was significantly less compared with that from S908.D2 parental-bearing mice (parental; 29.8 u $ml^{-1}$ *vs* vp.2; 6.3 u $ml^{-1}$). To compensate for the decrease in IL-2 levels caused by the overproduction of $PGE_2$, the parental and progressed lines were treated with lentinan and IL-2. The results shown in Figure 2 demonstrate that this combination is fully effective for the S908.D2 parental line (data not shown) as well as the S908.D2-vp.2 line, resulting in a complete cure. IL-2 alone did not produce any significant anti-tumour effects in either S908.D2-vp.2 (Figure 2) or S908.D2 parental lines (data not shown). These results suggest that the augmented production of $PGE_2$ in progressed lines results in the acquisition of resistance to chemoimmunotherapy that consists of CY/5-FU/5'-DFUR and lentinan.

### Disussion

Many studies have speculated on the mechanisms involved in the acquisition of resistance to chemotherapeutic agents by tumour cells (Schimke, 1984; Bradly *et al.*, 1988; Gottesman, 1988; Kramer *et al.*, 1988). These studies reveal at least two types of drug resistance; one is quite specific for a selected agent, such as an increase in the amount of dihydrofolate reductase in methotrexate-selected cells, and the other is less specific to selecting agents, such as an increase in the level of P-glycoprotein (Schimke, 1984; Gottesman, 1988). The resistance observed in our system is distinct from the inherent resistance against selected chemotherapeutic agents because the progressed lines were developed during therapy with CY and lentinan and subsequently acquired resistance to therapy with 5-FU/5'-DFUR and lentinan (Table III and data not shown). Furthermore, the resistance is believed to be distinct from multidrug resistance, such as the increase in the level of P-glycoprotein, because sensitivity *in vitro* of the S908.D2 parental and S908.D2 vp.1–3 progressed lines to both 5-FU and 4-deoxycyclophosphamide did not change (Figure 1).

Several studies have shown that the loss of tumour-associated antigens and a decrease in the expression of MHC class I antigens on tumour cells results in the escape of tumour cells from surveillance by host immune cells (Doherty *et al.*, 1984; Tanaka and Tevethia, 1988). In the present study, both susceptibility to CTLs, NK cells, LAK cells or activated macrophages and expression of MHC class I antigens between parental and progressed lines did not vary (Table IV and data not shown). These results indicate that the acquired resistance of the progressed lines to chemoimmunotherapy was not due to decreased susceptibility to immune effector cells.

Many studies have also shown that tumour cell- or host-derived typical immunosuppressive factors participate in the depression of host immunity against tumours and in the subsequent enhancement of tumour growth and metastases (Ceuppens and Goodwin, 1981; Kehrl *et al.*, 1986). TGF-$\beta$ and $PGE_2$ are the immunosuppressive factor(s) indicated so far (Ceuppens and Goodwin, 1981; Kehrl *et al.*, 1986; Li *et al.*, 1993). In the present study using an S908.D2 murine fibrosarcoma, TGF-$\beta$ does not appear to be involved in the acquired resistance of the mice to therapeutic drugs (Table V). We do demonstrate, however, that $PGE_2$ production in the tumour cells increases gradually during repeated therapy (Table V). It is well known that $PGE_2$ inhibits both IL-2 production and IL-2 receptor expression on T cells (Krause and Deutsch, 1991; Rappaport and Dodge, 1982; Parhar and Lala, 1987). Furthermore, recent studies revealed that $PGE_2$ favours a Th2-type of immune response by inhibiting the production of IFN-$\gamma$ by Thl T cells (Phipps *et al.*, 1991). These findings suggest that the defect in the IL-2/IL-2 receptor system is a possible mechanism for the acquired resistance of progressed lines to chemoimmunotherapy. The production of IL-2 by spleen cells from S908.D2-vp.2-bearing mice was markedly decreased compared with that from the parental S908.D2-bearing mice. Previous studies from our laboratories have shown that lentinan is able to augment the responsiveness of immune effector cells to IL-2 but that lentinan did not augment the production of IL-2 (Hamuro and Chihara, 1985). Therefore, it may be possible to restore the depressed responsiveness to IL-2 by the application of lentinan *in vivo* and to compensate for the reduced production of IL-2 by the exogenous infusion of IL-2. The combination therapy of lentinan and IL-2 was completely effective against S908.D2-vp.2 (Figure 2). These data suggest that the increased production of $PGE_2$ and the resulting reduced production of IL-2 may be involved in the acquisition of resistance to chemoimmunotherapy in the progressed S908.D2 lines, although we cannot exclude the participation of TGF-$\beta$ or other soluble factors in different model systems.

Several mechanisms may be responsible for the augmented production of $PGE_2$ from progressed lines. Tumour cells that produce high amounts of $PGE_2$ have been reported to have an increase in metastatic properties relative to tumour cells that produce less $PGE_2$ (Mahan *et al.*, 1985). Since S908.D2-vp.1 to vp.3 (progressed tumour lines) were established from metastatic cells in the lymph node, clones producing increased levels of $PGE_2$ might have been selected for. This possibility may be excluded, however, because metastatic lines (S908.D2-nt.1, -nt.2 or -nt.3) from the lymph nodes of mice that did not received therapy did not acquire resistance to the same chemoimmunotherapy and did not show enhanced $PGE_2$ production. Another possibility is that chemoimmunotherapy itself increases the production of $PGE_2$ in tumour cells. Since the anti-tumour effects of chemoimmunotherapy with CY and lentinan are T cell dependent (M Suzuki *et al.*, unpublished results), host inflammatory cells may be involved in the tumour disruption process. Recent reports demonstrated that oxygen radicals produced by host inflammatory cells induced the augmentation of $PGE_2$ production by tumour cells and that the enhanced production of $PGE_2$ from tumour cells is closely related to the phenotype changes of tumour cells from benign to malignant (Okada *et al.*, 1990, 1992, 1994). The elucidation of exact mechanisms for the increased production of $PGE_2$ during therapy requires further detailed studies. In summary, the results presented here highlight the importance of elucidating the biochemical pathways leading to augmented $PGE_2$ production during chemoimmunotherapy in order to understand the mechanisms of tumour progression.

The findings described in this paper demonstrate that tumour cell progression is also induced in chemoimmunotherapy, as it is in chemotherapy. The elucidation of the regulatory mechanisms of tumour cell progression *in vivo* during chemoimmunotherapy may result in new methods for the treatment of cancer patients, such as combination therapy with lentinan and IL-2.

**Acknowledgements**
We thank Dr S Fujimoto and Dr H Fujiwara for providing us with S908.D2 fibrosarcoma and MvlLu cells respectively. We also thank Dr T Yoshioka for providing us with 4-deoxycyclophosphamide.

### References

BALCH CM, DOUGHERTY PA, CLOUD GA AND TILDEN AB. (1984). Prostaglandin E2-mediated suppression of cellular immunity in colon cancer patients. *Surgery*, **95**, 71–77.

BRADLY G, JURANKA P AND LING V. (1988). Mechanisms of multidrug resistance. *Biochim. Biophys. Acta*, **948**, 87–128.

CEUPPENS J AND GOODWIN J. (1981). Prostaglandins and the immune response to cancer (review). *Anticancer Res.*, **1**, 71–78.

CHANG KC AND LOEB LA. (1993). Genomic instability and tumour progression: mechanistic considerations. *Adv. Cancer Res.*, **60**, 121–156.

CHEIFETZ S, WEATHERBEE JA, TSANG ML, ANDERSON JK, MOLE JE, LUCAS R AND MASSAGUE J. (1987). The transforming growth factor β system, a complex pattern of cross-reactive ligands and receptors. *Cell*, **48**, 409–415.

CHIHARA G, HAMURO J, MAEDA YY, ARAI Y AND FUKUOKA F. (1969). Fractionation and purification of the polysaccharides with marked anti-tumor activity, especially lentinan, from *Lentinus edodes* (Berk.) Sing. (an edible mushroom). *Cancer Res.*, **30**, 2776–2781.

DOHERTY PC, KNOWLES BB AND WETTSTEIN PJ. (1984). Immunological surveillance of tumors in the context of major histocompatibility complex restriction of T cell function. *Adv. Cancer Res.*, **42**, 1–65.

GOLDIE JH AND COLDMAN AJ. (1979). A mathematic model for relating the drug sensitivity of tumors to their spontaneous mutation rate. *Cancer Treat. Rep.*, **63**, 1727–1733.

GOTTESMAN MM. (1988). The multidrug transporter, a double-edged sword. *J. Biol. Chem.*, **263**, 12163–12166.

HAMURO J AND CHIHARA G. (1985). Lentinan, a T-cell-oriented immunopotentiator: its experimental and clinical applications and possible mechanisms of immune modulation. In *Immune Modulation Agents and their Mechanisms*. Fenicle RL (ed) pp. 409–437. Dekker.: New York.

HUNTER T. (1991). Cooperation between oncogenes. *Cell*, **64**, 249–270.

IMAMURA F, HORAI T, MUKAI M, SHINKAI K AND AKEDO H. (1990). Potentiation of invasive capacity of rat ascites hepatoma cells by adriamycin. *Cancer Res.*, **50**, 2018–2021.

JOHNSON JP, RIETHMULLER G AND SCHIRRMACHER V. (1989). Tumor immunology: Paul Ehrlich heritage. *Immunol. Today*, **10**, S35–S37.

KEHRL JH, WAKEFIELD LM, ROBERTS AB, JAKOWLEW S, ALVAREZ-MON M, DERYNCK R, SPORN MB AND FAUCI AS. (1986). Production of transforming growth factor β by human T lymphocytes and its potential role in the regulation of T cell growth. *J. Exp. Med.*, **163**, 1037–1050.

KRAMER RA, ZAKHER J AND KIM J. (1988). Role of the glutathione redox cycle in acquired and *de novo* multidrug resistance. *Science*, **241**, 694–697.

KRAUSE DS AND DEUTSCH C. (1991). Cyclic AMP directly inhibits IL-2 receptor expression in human T cells: expression of both p55 and p75 subunits is affected. *J. Immunol.*, **146**, 2285–2294.

LI X-F, TAKIUCHI H, ZOU J-P, KATAGIRI T, YAMAMOTO N, NAGATA T, ONO S, FUJIWAHA H AND HAMAOKA T. (1993). Transforming growth factpr-β (TGF-β)-mediated immunosuppression in the tumor-bearing state: enhanced production of TGF-β and a progressive increase in TGF-β susceptibility of anti-tumour $CD4^+$ T cell function. *Jpn. J. Cancer Res.*, **84**, 315–325.

MCMILLAN TJ AND HART IR. (1987). Can cancer chemotherapy enhance the malignant behavior of tumours? *Cancer Metastasis Rev.*, **6**, 503–520.

MAHAN M, MEUNIER J, NEWBY M AND YOUNG MR. (1985). Prostaglandin E2 production by EL 4 leukemia cells from C57BL/6 mice: mechanism for tumor dissemination. *J. Natl Cancer Inst.*, **74**, 191–195.

MITCHELL MS. (1992). Chemotherapy in combination with biomodulation: A 5-year experience with cyclophosphamide and interleukin-2. *Semin. Oncol.*, **19**, 80–87.

MOSMANN T. (1983). Rapid colorimetric assay for cellular growth and survival: application to proliferation and cytotoxicity assays. *J. Immunol. Methods*, **65**, 55–63.

OKADA F, HOSOKAWA M, HASEGAWA J, ISHIKAWA M, CHIBA I, NAKAMURA Y AND KOBAYASHI H. (1990). Regression mechanisms of mouse fibrosarcoma cells after in vitro exposure to quercetin: diminution of tumorigenicity with a corresponding decrease in the production of prostaglandin E2. *Cancer Immunol. Immunother.*, **31**, 358–364.

OKADA F, HOSOKAWA M, HAMADA J-I, HASEGAWA J, KATO M, MIZUTANI M, REN J, TAKEICHI N AND KOBAYASHI H. (1992). Malignant progression of a mouse fibrosarcoma by host cells reactive to a foreign body (gelatin sponge). *Br. J. Cancer*, **66**, 635–639.

OKADA F, HOSOKAWA M, HASEGAWA J, KURAMITSU Y, NAKAI L, LAO H, KOBAYASHI H AND TAKEICHI N. (1994). Enhancement of *in vitro* prostaglandin E2 production by mouse fibrosarcoma cells after co-culture with various anti-tumour effector cells. *Br. J. Cancer*, **70**, 233–238.

PACIUCCI PA, HOLLAND JF, RYDER JS, KONEFAL RG, BEKESI GJ, ODCHIMAR R AND GORDON R. (1989). Immunotherapy with interleukin-2 by constant infusion with and without adoptive transfer and weekly doxorubicin. *Cancer Treat. Rev.*, **16**, 67–81.

PARHAR RS AND LALA PK. (1987). Amelioration of B16F10 melanoma lung metastasis in mice by a combination therapy with indomethacin and interleukin 2. *J. Exp. Med.*, **165**, 14–28.

PHIPPS RP, STEIN SH AND ROPER RL. (1991). A new view of prostaglandin E regulation of the immune response. *Immunol. Today*, **12**, 349–352.

RAPPAPORT RS AND DODGE GR. (1982). Prostaglandin E inhibits the production of human interleukin 2. *J. Exp. Med.*, **155**, 943–948.

SATOH T, MATSUI H, SHIBAHARA S, KOBAYASHI T, MORINAGA Y, KASHIMA N, YAMASAKI S, HAMURO J AND TANIGUCHI T. (1987). New approaches for the high-level expression of human interleukin-2 cDNA in *Escherichia coli*. *J. Biochem.*, **101**, 525–534.

SCHIMKE RT. (1984). Gene amplification, drug resistance, and cancer. *Cancer Res.*, **44**, 1735–1742.

SUZUKI M, TAKATSUKI F, MAEDA YY, HAMUROA J AND CHIHARA G. (1994). Anti-tumor and immunological activity of lentinan in comparison with LPS. *Int. J. Immunopharmacol.*, **16**, 462–468.

TADA T, OHZEKI S, UTSUMI K, TAKIUCHI H, MURAMATSU M, LI X-F, SHIMIZU J, FUJIWARA H AND HAMAOKA T. (1991). Transforming growth factor β-induced inhibition of T cell function: susceptibility difference in T cells of various phenotypes and functions and its relevance to immunosuppression in the tumor-bearing state. *J. Immunol.*, **146**, 1077–1082.

TAGUCHI T, FURUE H, KIMURA T, KONDOH T, HATTORI T, ITO I AND OGAWA N. (1985). Endpoint result of a randomized controlled study on the treatment of gastro-intestinal cancer with a combination of lentinan and chemotherapeutic agents. In *Rationale of Biological Response Modifiers in Cancer Treatment*. Tsubura E, Urushizaki I and Aoki T (eds) pp.151–166. Excerpta Medica: Amsterdam.

TANAKA Y AND TEVETHIA SS. (1988). *In vitro* selection of SV40 T antigen epitope loss variants by site-specific cytotoxic T lymphocyte clones. *J. Immunol.*, **140**, 4348–4354.

TURVER CJ AND BROWN RC. (1987). The role of catalytic iron in asbestos induced lipid peroxidation and DNA-strand breakage in C3H10T1/2 cells. *Br. J. Cancer*, **56**, 133–136.

VOGELSTEIN B, FEARON ER, KERN SE, HAMILTON SR, PREISINGER AC, NAKAMURA Y AND WHITE R. (1989). Allelotype of colorectal carcinomas. *Science*, **244**, 207–211.

WARD JF. (1988). DNA damage produced by ionizing radiation in mammalian cells: Identities, mechanisms of formation and repairability. *Prog. Nucleic Acid Res.*, **35**, 95–125.

ZIMMARMAN R AND CERUTTI PA. (1984). Active oxygen acts as a promotor of transformation in mouse embryo C3H/10T1/2C18 fibroblast. *Proc. Natl Acad. Sci. USA*, **81**, 2085–2087.


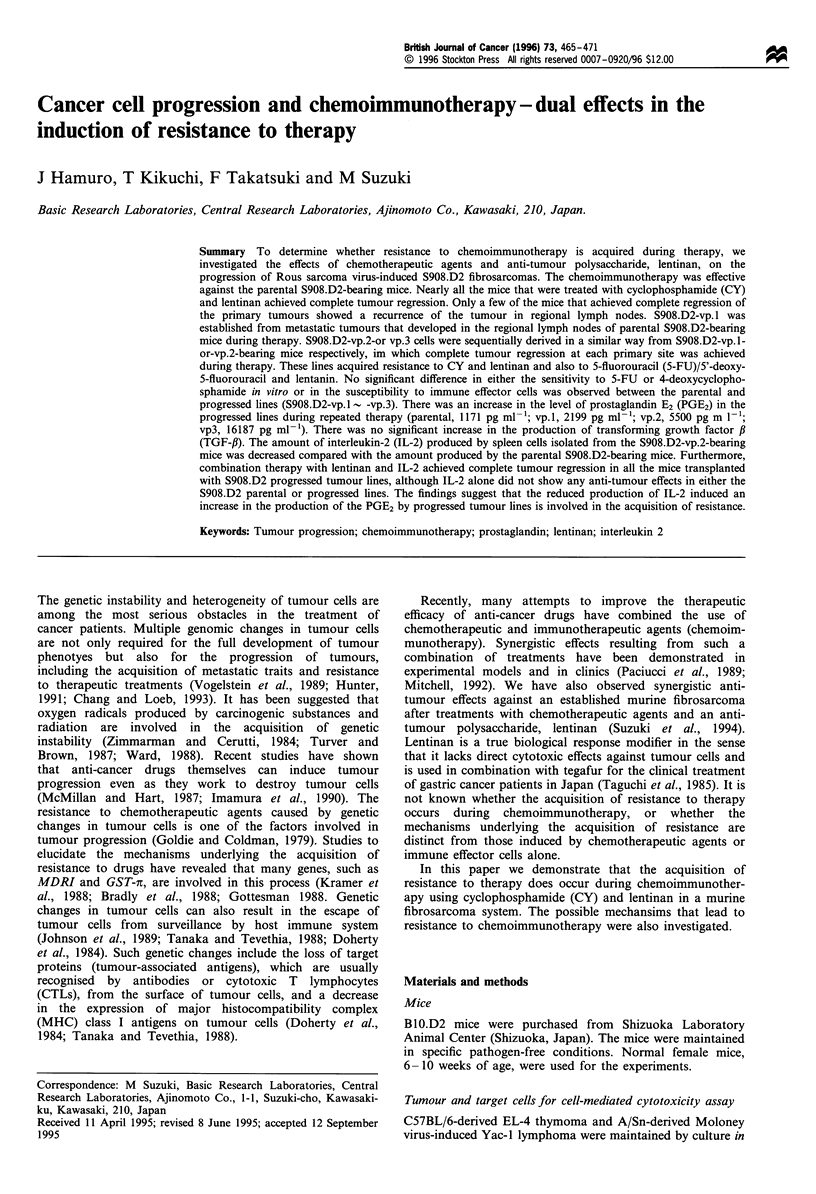

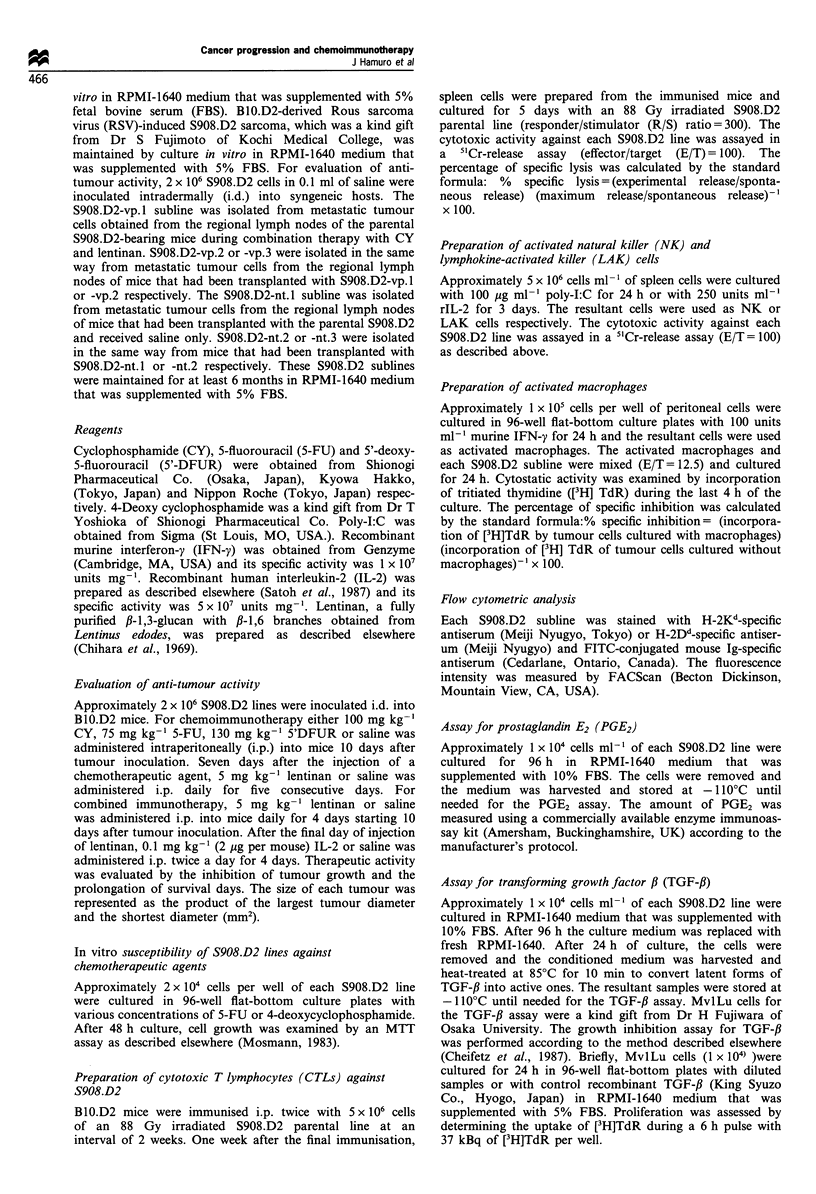

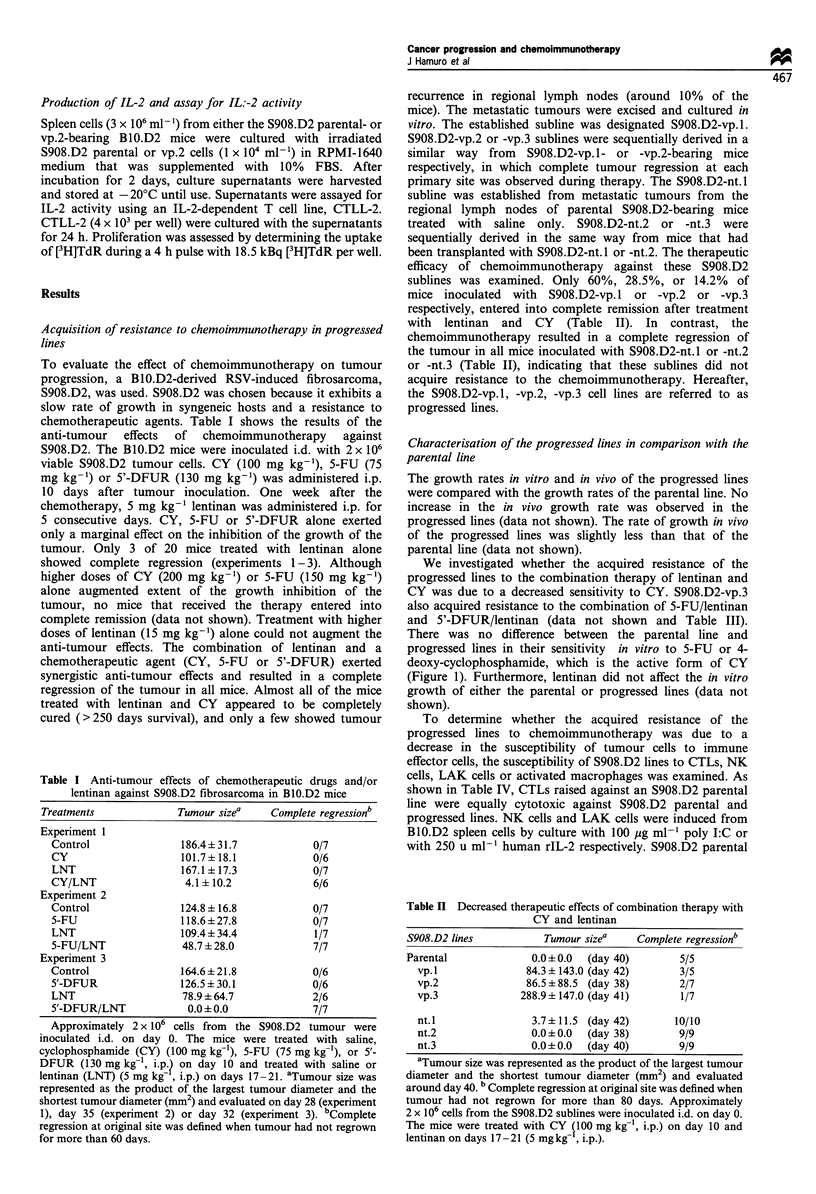

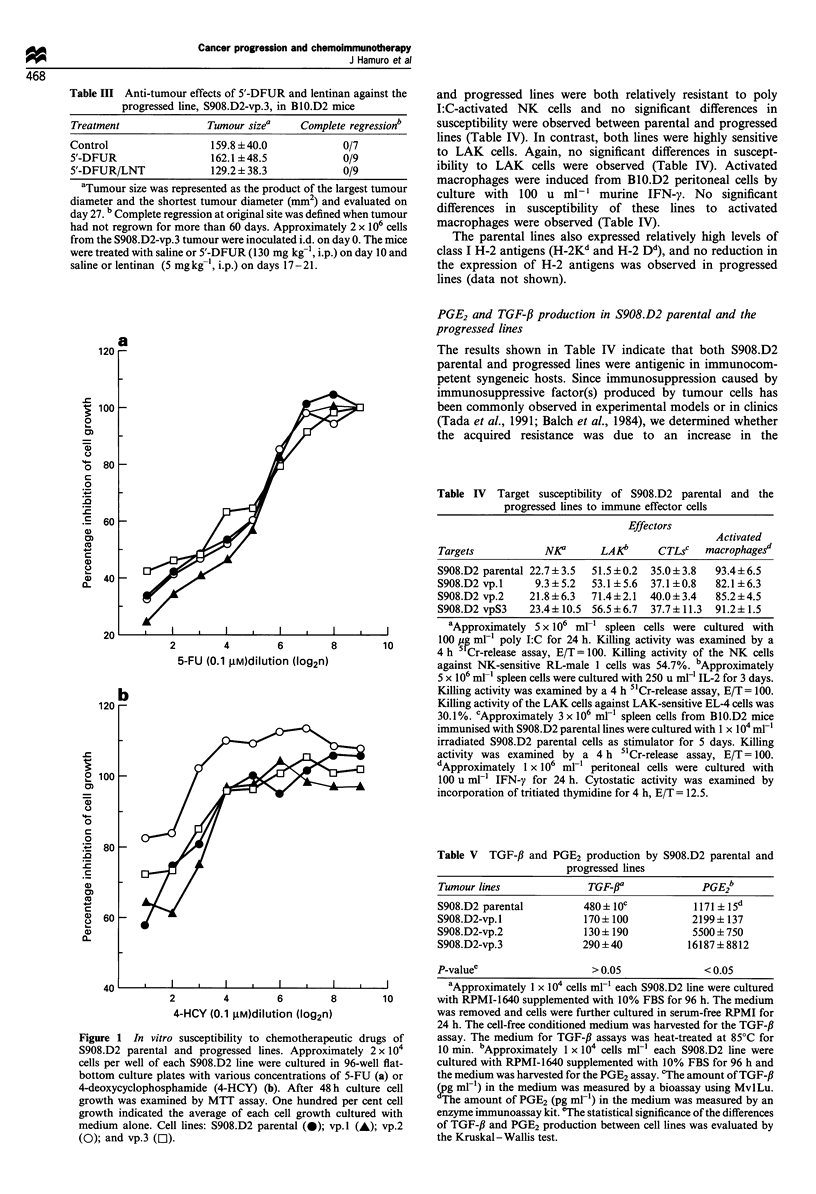

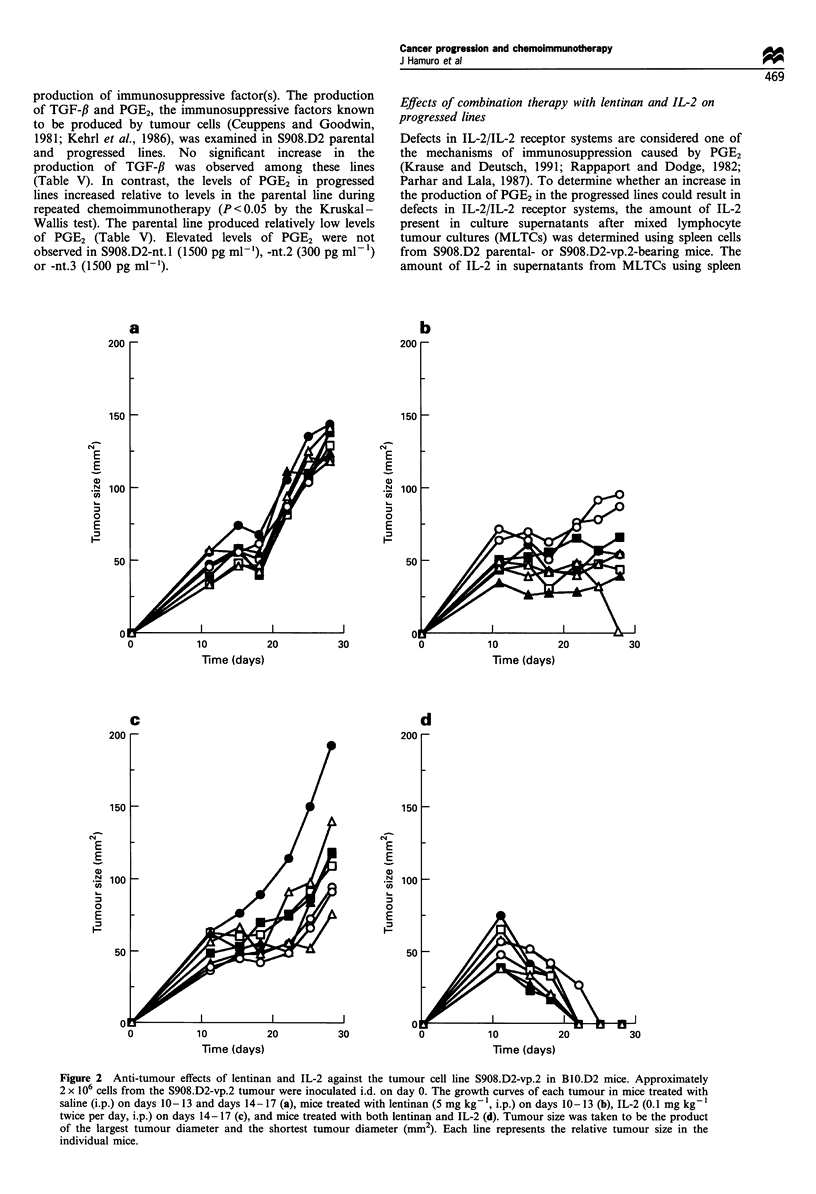

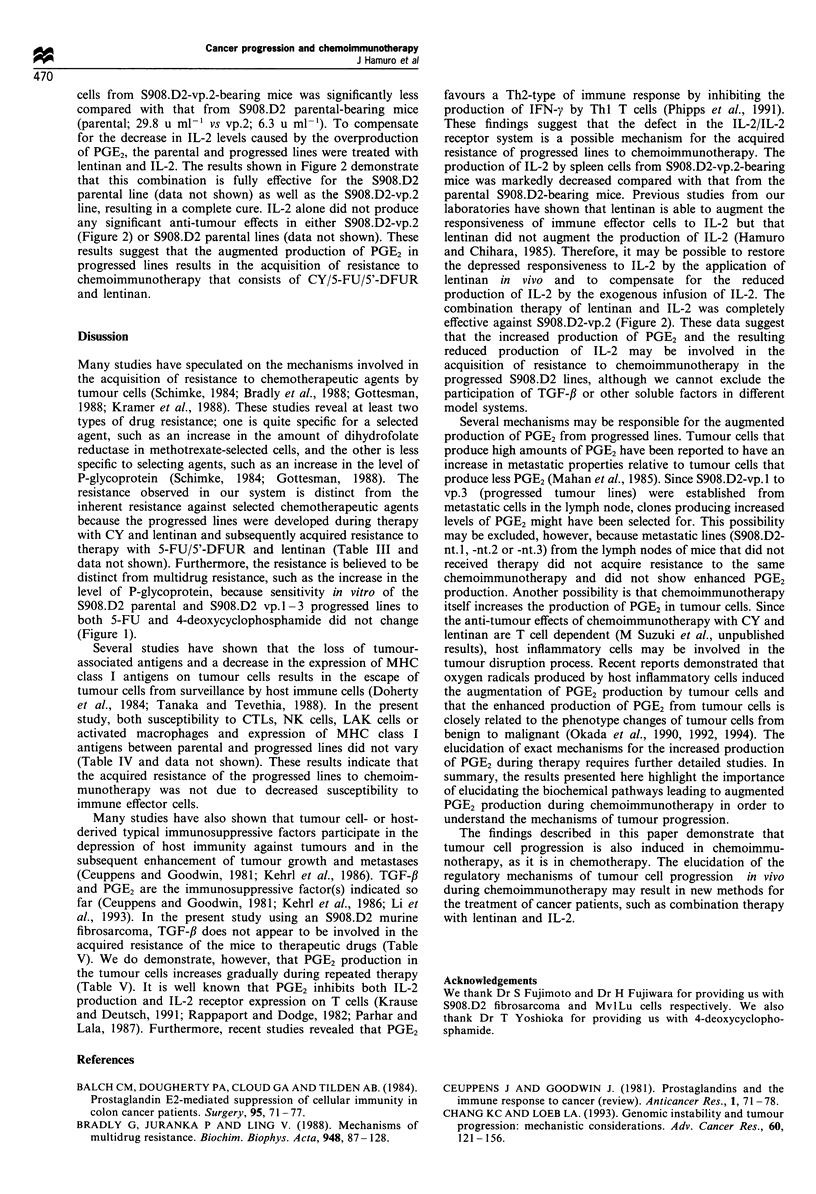

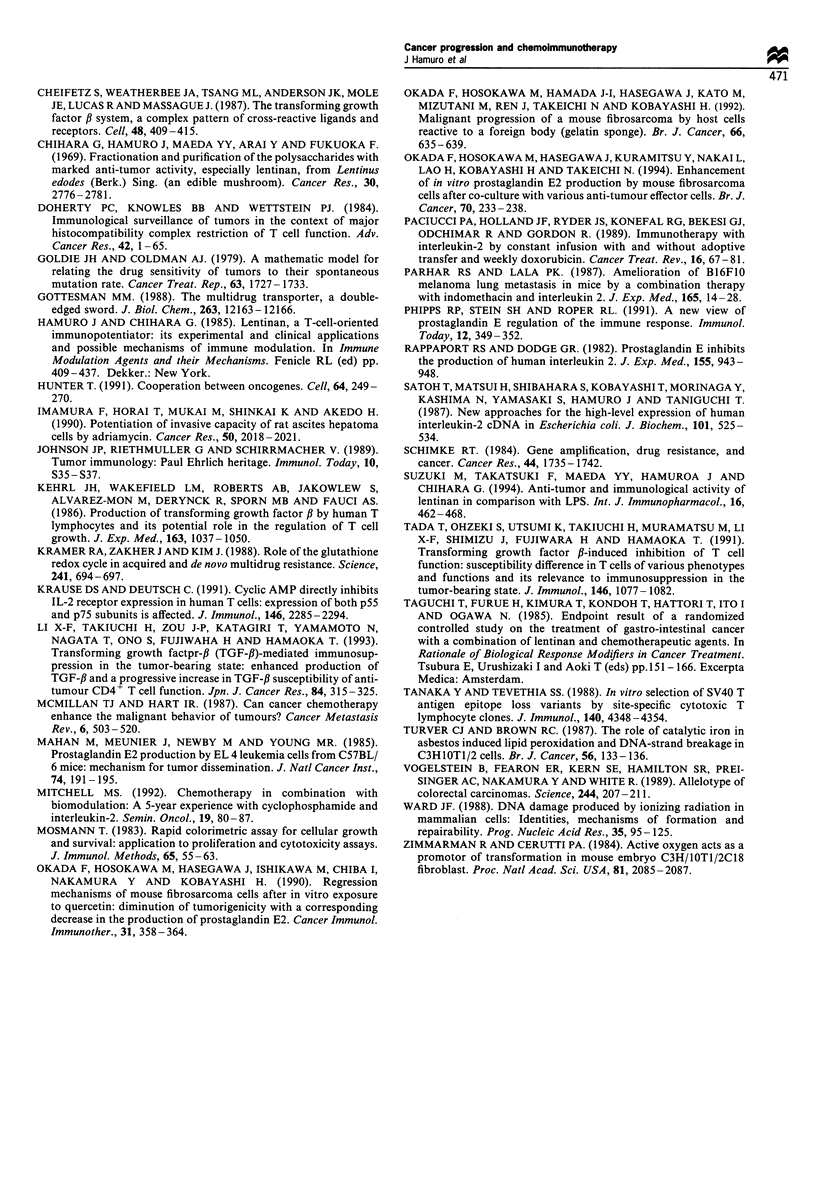


## References

[OCR_00926] Balch C. M., Dougherty P. A., Cloud G. A., Tilden A. B. (1984). Prostaglandin E2-mediated suppression of cellular immunity in colon cancer patients.. Surgery.

[OCR_00929] Bradley G., Juranka P. F., Ling V. (1988). Mechanism of multidrug resistance.. Biochim Biophys Acta.

[OCR_00933] Ceuppens J., Goodwin J. (1981). Prostaglandins and the immune response to cancer (review).. Anticancer Res.

[OCR_00950] Cheifetz S., Weatherbee J. A., Tsang M. L., Anderson J. K., Mole J. E., Lucas R., Massagué J. (1987). The transforming growth factor-beta system, a complex pattern of cross-reactive ligands and receptors.. Cell.

[OCR_00937] Cheng K. C., Loeb L. A. (1993). Genomic instability and tumor progression: mechanistic considerations.. Adv Cancer Res.

[OCR_00955] Chihara G., Hamuro J., Maeda Y., Arai Y., Fukuoka F. (1970). Fractionation and purification of the polysaccharides with marked antitumor activity, especially lentinan, from Lentinus edodes (Berk.) Sing. (an edible mushroom).. Cancer Res.

[OCR_00962] Doherty P. C., Knowles B. B., Wettstein P. J. (1984). Immunological surveillance of tumors in the context of major histocompatibility complex restriction of T cell function.. Adv Cancer Res.

[OCR_00966] Goldie J. H., Coldman A. J. (1979). A mathematic model for relating the drug sensitivity of tumors to their spontaneous mutation rate.. Cancer Treat Rep.

[OCR_00973] Gottesman M. M., Pastan I. (1988). The multidrug transporter, a double-edged sword.. J Biol Chem.

[OCR_00982] Hunter T. (1991). Cooperation between oncogenes.. Cell.

[OCR_00988] Imamura F., Horai T., Mukai M., Shinkai K., Akedo H. (1990). Potentiation of invasive capacity of rat ascites hepatoma cells by adriamycin.. Cancer Res.

[OCR_00993] Johnson J. P., Riethmüller G., Schirrmacher V. (1989). Tumor immunology: Paul Ehrlich's heritage.. Immunol Today.

[OCR_00999] Kehrl J. H., Wakefield L. M., Roberts A. B., Jakowlew S., Alvarez-Mon M., Derynck R., Sporn M. B., Fauci A. S. (1986). Production of transforming growth factor beta by human T lymphocytes and its potential role in the regulation of T cell growth.. J Exp Med.

[OCR_01005] Kramer R. A., Zakher J., Kim G. (1988). Role of the glutathione redox cycle in acquired and de novo multidrug resistance.. Science.

[OCR_01010] Krause D. S., Deutsch C. (1991). Cyclic AMP directly inhibits IL-2 receptor expression in human T cells: expression of both p55 and p75 subunits is affected.. J Immunol.

[OCR_01015] Li X. F., Takiuchi H., Zou J. P., Katagiri T., Yamamoto N., Nagata T., Ono S., Fujiwara H., Hamaoka T. (1993). Transforming growth factor-beta (TGF-beta)-mediated immunosuppression in the tumor-bearing state: enhanced production of TGF-beta and a progressive increase in TGF-beta susceptibility of anti-tumor CD4+ T cell function.. Jpn J Cancer Res.

[OCR_01025] Mahan M., Meunier J., Newby M., Young M. R. (1985). Prostaglandin E2 production by EL 4 leukemia cells from C57BL/6 mice: mechanism for tumor dissemination.. J Natl Cancer Inst.

[OCR_01020] McMillan T. J., Hart I. R. (1987). Can cancer chemotherapy enhance the malignant behaviour of tumours?. Cancer Metastasis Rev.

[OCR_01033] Mitchell M. S. (1992). Chemotherapy in combination with biomodulation: a 5-year experience with cyclophosphamide and interleukin-2.. Semin Oncol.

[OCR_01038] Mosmann T. (1983). Rapid colorimetric assay for cellular growth and survival: application to proliferation and cytotoxicity assays.. J Immunol Methods.

[OCR_01049] Okada F., Hosokawa M., Hamada J. I., Hasegawa J., Kato M., Mizutani M., Ren J., Takeichi N., Kobayashi H. (1992). Malignant progression of a mouse fibrosarcoma by host cells reactive to a foreign body (gelatin sponge).. Br J Cancer.

[OCR_01044] Okada F., Hosokawa M., Hasegawa J., Ishikawa M., Chiba I., Nakamura Y., Kobayashi H. (1990). Regression mechanisms of mouse fibrosarcoma cells after in vitro exposure to quercetin: diminution of tumorigenicity with a corresponding decrease in the production of prostaglandin E2.. Cancer Immunol Immunother.

[OCR_01059] Okada F., Hosokawa M., Hasegawa J., Kuramitsu Y., Nakai K., Yuan L., Lao H., Kobayashi H., Takeichi N. (1994). Enhancement of in vitro prostaglandin E2 production by mouse fibrosarcoma cells after co-culture with various anti-tumour effector cells.. Br J Cancer.

[OCR_01066] Paciucci P. A., Holland J. F., Ryder J. S., Konefal R. G., Bekesi G. J., Odchimar R., Gordon R. (1989). Immunotherapy with interleukin-2 by constant infusion with and without adoptive cell transfer and with weekly doxorubicin.. Cancer Treat Rev.

[OCR_01070] Parhar R. S., Lala P. K. (1987). Amelioration of B16F10 melanoma lung metastasis in mice by a combination therapy with indomethacin and interleukin 2.. J Exp Med.

[OCR_01075] Phipps R. P., Stein S. H., Roper R. L. (1991). A new view of prostaglandin E regulation of the immune response.. Immunol Today.

[OCR_01078] Rappaport R. S., Dodge G. R. (1982). Prostaglandin E inhibits the production of human interleukin 2.. J Exp Med.

[OCR_01086] Sato T., Matsui H., Shibahara S., Kobayashi T., Morinaga Y., Kashima N., Yamasaki S., Hamuro J., Taniguchi T. (1987). New approaches for the high-level expression of human interleukin-2 cDNA in Escherichia coli.. J Biochem.

[OCR_01092] Schimke R. T. (1984). Gene amplification, drug resistance, and cancer.. Cancer Res.

[OCR_01094] Suzuki M., Takatsuki F., Maeda Y. Y., Hamuro J., Chihara G. (1994). Antitumor and immunological activity of lentinan in comparison with LPS.. Int J Immunopharmacol.

[OCR_01103] Tada T., Ohzeki S., Utsumi K., Takiuchi H., Muramatsu M., Li X. F., Shimizu J., Fujiwara H., Hamaoka T. (1991). Transforming growth factor-beta-induced inhibition of T cell function. Susceptibility difference in T cells of various phenotypes and functions and its relevance to immunosuppression in the tumor-bearing state.. J Immunol.

[OCR_01119] Tanaka Y., Tevethia S. S. (1988). In vitro selection of SV40 T antigen epitope loss variants by site-specific cytotoxic T lymphocyte clones.. J Immunol.

[OCR_01124] Turver C. J., Brown R. C. (1987). The role of catalytic iron in asbestos induced lipid peroxidation and DNA-strand breakage in C3H10T1/2 cells.. Br J Cancer.

[OCR_01127] Vogelstein B., Fearon E. R., Kern S. E., Hamilton S. R., Preisinger A. C., Nakamura Y., White R. (1989). Allelotype of colorectal carcinomas.. Science.

[OCR_01132] Ward J. F. (1988). DNA damage produced by ionizing radiation in mammalian cells: identities, mechanisms of formation, and reparability.. Prog Nucleic Acid Res Mol Biol.

[OCR_01137] Zimmerman R., Cerutti P. (1984). Active oxygen acts as a promoter of transformation in mouse embryo C3H/10T1/2/C18 fibroblasts.. Proc Natl Acad Sci U S A.

